# A New Era in Engineering Plastics: Compatibility and Perspectives of Sustainable Alipharomatic Poly(ethylene terephthalate)/Poly(ethylene 2,5-furandicarboxylate) Blends

**DOI:** 10.3390/polym13071070

**Published:** 2021-03-29

**Authors:** Dimitrios G. Papageorgiou, Irini Tsetsou, Raphael O. Ioannidis, George N. Nikolaidis, Stylianos Exarhopoulos, Nejib Kasmi, Dimitrios N. Bikiaris, Dimitris S. Achilias, George Z. Papageorgiou

**Affiliations:** 1 School of Engineering and Materials Science, Queen Mary University of London, Mile End Road, London E1 4NS, UK; 2 Chemistry Department, University of Ioannina, P.O. Box 1186, 45110 Ioannina, Greece; eirinitsetsou789@yahoo.gr (I.T.); pch01348@uoi.gr (R.O.I.); geoniknikolaidis@gmail.com (G.N.N.); 3 Department of Food Science and Technology, International Hellenic University, P.O. Box 141, 57400 Thessaloniki, Greece; stelexar@yahoo.gr; 4 Department of Materials Research and Technology (MRT), Luxembourg Institute of Science and Technology (LIST), 5 Avenue des Hauts-Fourneaux, L-4362 Esch-sur-Alzette, Luxembourg; nejibkasmi@gmail.com; 5 Laboratory of Polymer Chemistry and Technology, Department of Chemistry, Aristotle University of Thessaloniki, 54124 Thessaloniki, Greece; dbic@chem.auth.gr (D.N.B.); axilias@chem.auth.gr (D.S.A.); 6 Institute of Materials Science and Computing, University Research Center of Ioannina (URCI), 45110 Ioannina, Greece

**Keywords:** poly(ethylene furanoate), poly(ethylene terephthalate), blends, crystallization

## Abstract

The industrialisation of poly(ethylene 2,5-furandicarboxylate) for total replacement of poly(ethylene terephthalate) in the polyester market is under question. Preparation of high-performing polymer blends is a well-established strategy for tuning the properties of certain homopolymers and create tailor-made materials to meet the demands for a number of applications. In this work, the structure, thermal properties and the miscibility of a series of poly(ethylene terephthalate)/poly(ethylene 2,5-furandicarboxylate) (PET/PEF) blends have been studied. A number of thermal treatments were followed in order to examine the thermal transitions, their dynamic state and the miscibility characteristics for each blend composition. Based on their glass transition temperatures and melting behaviour the PET/PEF blends are miscible at high and low poly(ethylene terephthalate) (PET) contents, while partial miscibility was observed at intermediate compositions. The multiple melting was studied and their melting point depression was analysed with the Flory-Huggins theory. In an attempt to further improve miscibility, reactive blending was also investigated.

## 1. Introduction

Not surprisingly, over the last decade there is growing interest in the preparation of new chemicals and materials based on renewable resources; biomass-derived fuel and chemicals are a promising alternative to fossil based materials [1,2]. Generally, bio-based plastics can be manufactured via three main pathways [3]. The first pathway involves the modification of natural polymers (e.g., for production of starch and cellulose-based plastics). The second—and most important—approach involves conversion of biomass to bio-based precursors (monomers) via biochemical and/or chemical transformation followed by polymerization of the monomers. As a result, conventional monomers (drop-in) and then conventional plastics, such as polyethylene (PE) or poly(ethylene terephthalate) (PET), can be produced. New monomers, especially aromatic ones, and new polymers such as poly(ethylene 2,5-furandicarboxylate) (PEF) can be also manufactured and are expected to have a key role in the new era of sustainable engineering plastics from biomass for the development of a biobased economy. Finally, the third approach involves polymers that can be formed from microorganisms such as (polyhydroxyalkanoates) (PHA), or from plants and finally by using CO_2_ as a feedstock [4,5,6,7].

2,5-furandicarboxylic acid is considered as one of the most important building blocks or top value-added chemicals derived from biomass, according to the U.S. Department of Energy [8,9]. FDCA can be used for the production of polyesters bearing furan moieties such as poly(ethylene 2,5-furan dicarboxylate) (PEF) and poly(butylene 2,5-furan dicarboxylate) (PBF) which can be regarded as the biobased alternatives of terephthalates [10,11,12,13]. PEF is the most important furan-based polyester as it can be formulated in films, fibers and bottles. It has been shown that PEF bottles exhibit 11 times better O_2_ barrier than their PET counterparts, 19 times better CO_2_ barrier and 1.6 times higher tensile modulus [14,15,16]. The improved properties of PEF are related with the differences in the stiffness and the motional dynamics of the aromatic rings, which are present in the PEF structure. The hindrance of furan ring-flipping explains the significant reduction in oxygen diffusion coefficient and permeability as compared to PET. In addition, a recent investigation by dielectric spectroscopy and differential scanning calorimetry revealed the important role of the restricted amorphous fraction [17]. The latter is of key importance to the gas barrier and mechanical properties. It has also been reported that use of PEF may lead to a reduction of the non-renewable energy use of about 40% to 50% while greenhouse gas (GHG) emissions can also be lowered by about 50%, compared to PET [18].

Poly(ethylene terephthalate) on the other hand, is the most important thermoplastic polyester in terms of its commercial value, as it used in several applications and especially in soft drink containers. The overall consumption of PET was 100 million tons in 2016 and it currently grows by 4% per year [19]. However, since the majority of the feedstocks for PET are petroleum-based, a number of potential routes have been proposed to synthesize PET monomers from biomass. Academic and industrial researchers have made significant efforts to produce 100% renewable PET by using drop-in replacements of PET precursors from biomass [20,21,22]. Due to its commercial importance, PET is one of the most studied polymers, hence a variety of copolymers, blends, and composites based on PET have been investigated [23,24,25,26,27].

The modification and functionalization of polymeric materials can be achieved with a number of strategies, including the fabrication of polymer blends. From the aspect of crystallizability of the constituents, binary polymer blends can be classified into amorphous/amorphous, crystalline/amorphous, and crystalline/crystalline systems [28]. Most of the studies on polymer blends are focused on pairs of two amorphous polymers or mixtures in which one of the components is semicrystalline. On the other hand, semicrystalline/semicrystalline polymer pairs have received less attention. It is usually more difficult experimentally to investigate the miscibility (in the amorphous phase) of blends of which both constituents are semicrystalline polymers. Miscibility is relatively rare in polymer pairs because of the entropy of mixing for high molecular weight chains [29,30]. For PET, blending with other polymers to improve gas barrier properties, transparency, thermal resistance, and recyclability is of special importance for applications such as in soft drink containers. Transesterification reactions and formation of copolymers both play a crucial role in improving interfacial adhesion and homogenization in PET blends [31]. Thus, reactive blending offers another opportunity for arriving to increased compatibilization. As a number of obstacles may exist towards the industrialization of PEF, including discoloration, low thermal stability and slow crystallization compared to PET, a more careful strategy for the introduction of PEF in the marketplace and a progressive transition from PET to PEF should be drawn. It is important to point out that the two polymers, PEF and PET, are expected to meet in the recycling stream from municipal wastes, so the compatibility of the PET-PEF blends is of special importance.

In this work, a series of PET-PEF blends were prepared and studied by a number of characterization techniques. The presence of bio-based PEF in the produced blends could reduce the environmental impact from the extensive use of petroleum-originating terephthalic acid, while maintaining the recyclability of the final product. Since recent efforts on the mass production of PEF have materialized by large companies such as Avantium, the blends can be readily produced in an industrial scale. Herein, the miscibility and crystallization of the blends were studied by means of differential scanning calorimetry (DSC), temperature modulated DSC (MDSC), wide-angle X-ray diffractometry (WAXD), and polarized light microscopy (PLM). Finally, reactive blending was simulated within a DSC pan at elevated temperatures, in order to evaluate its potential as a way to achieve homogeneity in the PET-PEF blends.

## 2. Experimental

### 2.1. Materials

Dimethyl terephthalate (DMT) was obtained from Du Pont De Nemours Co. and 2,5-furan dicarboxylic acid (purum 97%) was purchased from Sigma Aldrich Co. (Darmstadt, Germany) Ethylene glycol and tetrabutyl titanate (TBT) catalyst of analytical grade were purchased also from Sigma Aldrich Co. (Darmstadt, Germany). All other materials and solvents used were of analytical grade.

### 2.2. Synthesis of 2,5-Dimethylfuran-Dicarboxylate (DMF)

For the synthesis of DMF, 15.6 g of 2,5-furandicarboxylic acid, 200 mL of methanol anhydrite and 2 mL of concentrated sulfuric acid was transferred into a random flask (500 mL) and the mixture was refluxed for 5 h. The excess of the methanol was distilled and the solution was filtered through a disposable Teflon membrane filter. During filtration dimethylester was precipitated as white powder and following cooling, 100 mL of distilled water was added. The dispersion was partially neutralized by adding Na_2_CO_3_ 5% *w*/*v* during stirring while pH was measured continuously. The white powder was filtered and the solid was washed several times with distilled water and dried. The isolated white methylester was recrystallized with a mixture of 50/50 *v*/*v* methanol/water. Following cooling, 2,5-dimethylfuran-dicarboxylate (DMF) was precipitated in the form of white needles. The reaction yield was calculated at 83%.

### 2.3. Polyester Synthesis

**Scheme 1:**
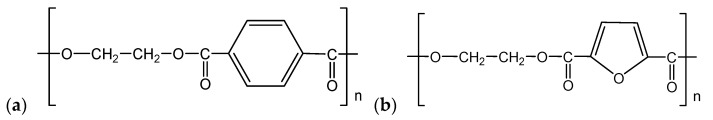
The chemical structures of poly(ethylene terephthalate) (PET) (**a**) and PEF (**b**).

The polyesters were prepared by the two-stage melt polycondensation method (esterification and polycondensation) in a glass batch reactor. For the preparation of PET (Scheme 1a) the proper amounts of DMT and EG at a molar ratio of diester/EG = 1/2.2 were charged into the reaction tube of the polyesterification apparatus. For the synthesis of PEF (Scheme 1b), a higher molar ratio was used (DMF/EG = 1/3). TBT (400 ppm) was added as catalyst and the apparatus with the reagents was evacuated several times and filled with argon in order to remove the whole oxygen amount. The reaction mixture was heated at 190 °C under argon atmosphere and stirring at a constant speed (350 rpm). For the synthesis of PEF the reagents were first heated at 160 °C under argon atmosphere for 2 h at 170 °C for additional 2 h and finally at 180–190 °C for 1 h. This first step (transesterification) is considered to complete after the collection of almost all the theoretical amount of CH_3_OH, which was removed from the reaction mixture by distillation and collected in a graduate cylinder.

In the second step of polycondensation, vacuum (5.0 Pa) was applied slowly over a period of time of about 30 min to remove the excess of diols and to avoid excessive foaming and furthermore to minimize oligomer sublimation, which is a potential problem during melt polycondensation. The temperature was gradually increased (1 h) for PET synthesis to 280 °C while stirring speed was increased at 720 rpm. The polycondensation continued for about 120 min at 280 °C. For PEF synthesis the temperature was slowly increased from 190 °C to 220 °C while stirring speed was increased at 720 rpm. The reaction continued at this temperature for 2 h and after that time the temperature was increased to 235 °C for 2 h and at 250 °C for additional 2 h. Following the completion of the polycondensation reaction, polyesters were easily removed, milled and washed with methanol.

### 2.4. Preparation of Blends

PET and PEF samples were synthesized by applying the melt polycondensation method. Then, PET-PEF blends were prepared by using the solvent method. A mixture of Chloroform/Trifluoroacetic acid 5/1 *v*/*v* was used as the mutual solvent of the two polymers. The blends were then removed from the solution as white powder by adding excess of methanol. The samples were annealed at 160 °C for 2 h prior to testing to enhance their crystallinity.

### 2.5. Polyester Characterization

#### 2.5.1. Wide Angle X-Ray Diffraction Patterns (WAXD)

X-ray diffraction measurements of the samples were performed using a MiniFlex II XRD system from Rigaku Co, with CuK_α_ radiation (λ = 0.154 nm) in the angle 2*θ* range from 5 to 60 degrees.

#### 2.5.2. Differential Scanning Calorimetry (DSC)

A Perkin–Elmer, Pyris Diamond DSC differential scanning calorimeter, calibrated with pure indium and zinc standards, was used. The system also included an Intracooler 2P cooling accessory, in order to achieve function at sub-ambient temperatures and high cooling rates. Samples of 5 ± 0.1 mg sealed in aluminium pans were used, to test the thermal behavior of the quenched polymers. In order to obtain amorphous materials, the samples were heated to 40 °C above the melting temperature (up to 280 °C) and held there for 2 min—in order to erase any thermal history—followed by rapid cooling with the highest achievable rate (nominal rate of 500 °C/min and real average rate of ~80 °C/min down to 0 °C). Isothermal crystallization experiments of the polymers at various temperatures below the melting point were performed. For the non-isothermal crystallization experiments, the samples were heated at temperatures higher than their equilibrium melting temperatures and then they were cooled at various cooling rates, ranging from 1.25 up to 10 °C/min. With regards to reactive blending, within an industrial environment it involves melt mixing in an extruder at temperatures higher than the melting temperatures of the elements of the blend. In order to simulate reactive blending in a much smaller scale this work, the blends were initially prepared from solution, as described above, and were subsequently melt-mixed inside the DSC pans. In detail, during reactive blending, the blends were heated at a rate of 20 °C/min up to a predetermined temperature that was well above the melting points of both components, where they were held for a specific time in each test before quenching to −30 °C. The quenched samples were subsequently heated again at 20 °C/min, starting from a temperature of at least 30 °C below the lower *T*_g_ of the polymers.

A TA Instruments modulated temperature DSC (MTDSC) TA Q2000, (New Castle, DE, USA) was also used for the temperature-modulated DSC measurements. The instrument was calibrated with indium for the heat flow and temperature, while the heat capacity was evaluated using sapphire standard. The sample mass was kept around 5 mg in all tests. The Al sample and reference pans are of identical mass with an error ± 0.01 mg. The temperature modulated DSC scans (TMDSC) were performed at a heating rate of 5 °C/min, with temperature modulation amplitude of 1 °C and a period of 60 s.

The modulated DSC measurements include the introduction of isothermal steps between the heating steps and the different signals correspond to different contributions during the heating process. The non-reversing signal is known to represent the melting of stacks of lamellae or separate polymer lamellae. The reversing signal is associated to the partial melting of the lamellae which recrystallize rapidly on existing signals. It enables us to distinguish events such as the glass temperature from other non-reversing processes, such as enthalpy relaxation or recrystallization, which are absolutely not present in the non-reversing signal. For this reason, the resolution of different thermal events with the use of modulated DSC measurements is standard practice in the study of the thermal properties of polymers.

#### 2.5.3. Polarizing Light Microscopy (PLM)

A polarizing light microscope (Nikon, Optiphot-2, Tokyo, Japan) equipped with a Linkam THMS 600 (Waterfield, Epsom, United Kingdom) heating stage, a Linkam TP 91 (Waterfield, Epsom, United Kingdom) control unit and also a Jenoptic ProgRes C10Plus camera (Jiena, Germany) with the Capture Pro 2.1 software was used for PLM observations.

## 3. Results and Discussion

### 3.1. Primary Characterisation

**Figure 1:**
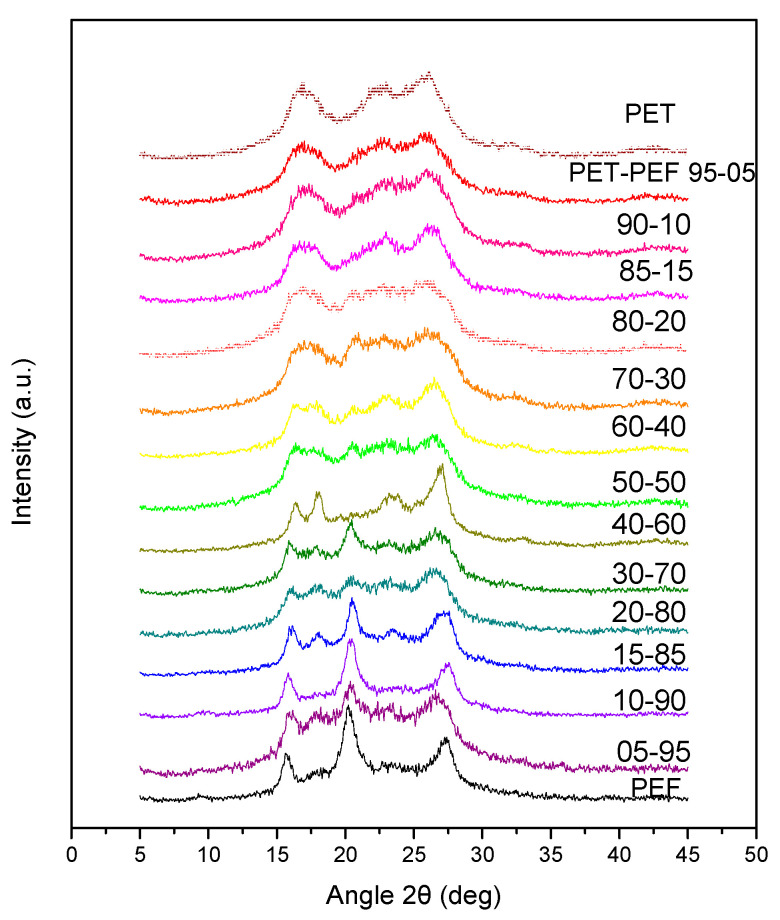
Wide-angle X-ray diffractometry (WAXD) patterns for the blends and for the PEF and PET homopolymers and their blends at different compositions as indicated.

The structure of the two homopolymers, PEF and PET was initially examined by WAXD (Figure 1). For PET, the thermodynamically stable a-form appeared from the characteristic WAXD pattern, with a triclinic unit cell with *a* = 4.56 Å, b = 5.94 Å, c = 10.75 Å, α = 98.5°, β = 118°, and γ = 112° [32]. The repeating unit along the c-axis contains one monomer, with the polymer chain tilted by ~5° with respect to the *c*-axis. For PEF unit cell the space group is P2_1_, with a monoclinic unit cell where *a* = 5.784 Å, *b* = 6.780 Å, *c* = 20.296 Å, and γ = 103.3°. The repeating unit consists of two monomers related to each other by a 2_1_ screw axis [33,34].

The WAXD patterns of the blends are shown in Figure 1. The peaks generally appear quite strong and pronounced, an indication of significant crystallinity, as a result of both solution crystallization and annealing procedures. Crystalline peaks for both polyesters can be detected in the patterns of the blends, especially for intermediate blend compositions. Based on this observation it can be realized that this can be considered as a crystalline/crystalline blend system [32,34,35].

**Figure 2:**
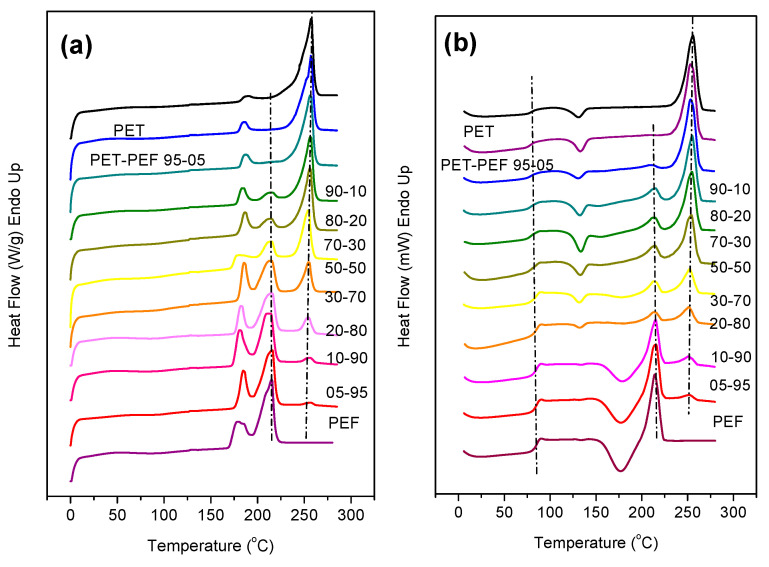
Differential scanning calorimetry (DSC) thermograms (**a**) for the semicrystalline blends recorded upon heating at 20 °C/min and (**b**) total Modulated Temperature DSC (MTDSC) signal for the fast-cooled samples upon heating at 5 °C/min.

The thermal transitions of the blends were studied by DSC. The DSC heating scans (at 20 °C/min) for the blends are shown in Figure 2a; melting peaks for both polymers are present, except for the PET-PEF 95-5 blend. In the thermograms of the latter, the melting of PEF is not clearly observed as a result of the low PEF content. A low temperature melting peak, just above 180 °C appears in all DSC thermograms, which should be associated mainly with the annealing process. Furthermore, it must be noted that for the blends, a slight decrease in the melting temperature of PET was recorded with increasing PEF content. This took place simultaneously with the reduction in the heat of fusion for the corresponding melting peaks, indicating lower degrees of crystallinity for intermediate blend compositions.

Figure 2b shows the total signal recorded from temperature modulated-DSC experiments for the blends, at a heating rate of 5 °C/min, after fast cooling at an initial rate of 80 °C/min in the DSC cell. MDSC is a powerful thermal analysis tool as it can separate reversible from non-reversible phenomena [36]. PEF is known to crystallize at relatively slow rates [37,38,39]. As it can be seen, only those blends with a PEF content higher than 80 wt.% were efficiently quenched and obtained in the amorphous state. PEF did cold-crystallize upon heating, but even cold-crystallization was suppressed with increasing the PET content beyond 10 wt.% in the blends. PET on the other hand crystallizes fast, so its crystallization cannot be efficiently suppressed upon cooling at a rate of 80 °C/min. Under these conditions, only a minor cold-crystallization peak can be detected and not pronounced cold crystallization. Melting peaks for both polymer components of the blends appeared, again with the exception of the PET-PEF 95-5 sample. The first dotted line in the figure at ~80 °C also indicates a glass temperature that will be further analyzed below. We should mention at this point the proximity of the glass temperatures for the two homopolymers: *T*_g(PET)_ = 81 °C and *T*_g(PEF)_ = 88 °C. This fact makes a systematic investigation of the thermodynamic state with respect to miscibility for the two components in the blends, a difficult task. 

**Figure 3:**
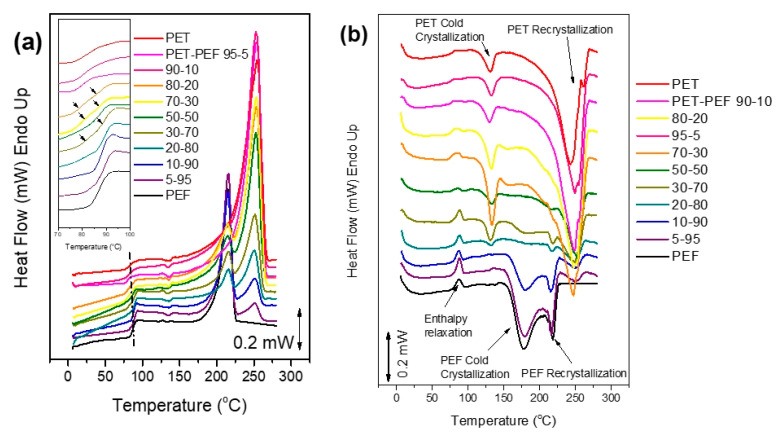
Modulated Temperature DSC (MDSC) thermograms for the fast-cooled upon heating at 5 °C/min: (**a**) reversing signals and (**b**) non-reversing signals.

The reversing signals from the MDSC curves of the blends were also studied (Figure 3a). The results showed a shift in the glass temperature region towards higher temperatures with increasing PEF content, and there were also indications for double *T_g_*s. In the non-revering signal curves (Figure 3b), besides the recrystallization exothermic peak in the melting temperature region, a cold-crystallization peak can be detected for PET and at slightly higher temperatures for PEF. According to Woo et al. [40], for polymer blends in which the *T_g_* values are very close, a single cold-crystallization peak is an indication of miscibility. Therefore, for the PET-PEF blends this prerequisite for miscibility seems that it cannot be satisfied. It should be stated though that in this work, detailed non-reversing signals recorded during slow heating scans (average heating rate 5 °C/min) from MDSC were used, in contrast to the work of Woo et al. where only standard DSC traces recorded at a fast heating rate of 20 °C/min were analyzed [40].

**Figure 4:**
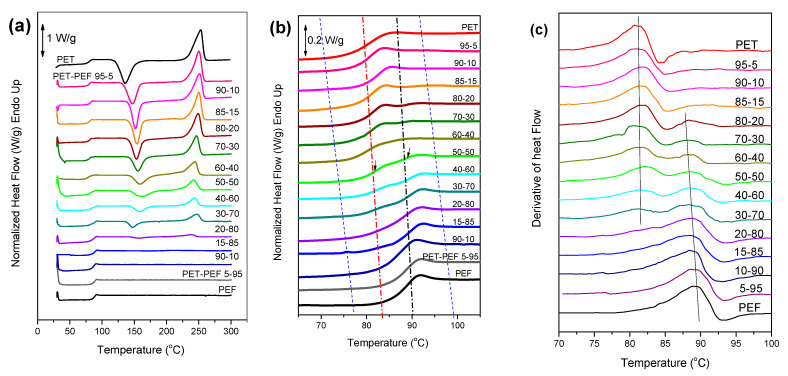
Power compensation DSC thermograms of melt quenched blends at 20 °C/min (**a**) full temperature scale and (**b**) details of the glass transition temperature range; the black dotted line corresponds to the *T*_g_ of PEF and the red dotted line corresponds to the *T*_g_ of PET. (**c**) First derivative of the heat flow plotted as a function of temperature for several PET/PEF compositions. Red and blue arrows indicate glass temperatures of PEF and PET segments, respectively.

A high-sensitivity power-compensation DSC was also elaborated for the study of the glass temperatures in the blends. The samples were first melt quenched on an ice-cold metal plate. Effective quenching resulted in almost completely amorphous samples as it can be concluded from the comparison between the heat of cold crystallization and the heat of the subsequent melting during the DSC scans at 20 °C/min (Figure 4a), which were practically equal. As it can be seen in Figure 4b where the details of the glass temperatures are shown, two separate glass temperatures can be seen at several compositions, as pointed out with the two arrows. The red dotted line in the figure correspond to the glass temperature of PET and the black dotted line shows the *T*_g_ of PEF. It is known that thermodynamic miscibility cannot be concluded on the basis of a dual *T*_g_ as both immiscible and miscible blends exhibit two *T*_g_s. From Figure 4b, significant windows of miscibility can be observed at compositions ranging from slightly less than 100% PET to 85% PET (e.g., 0–15% PEF in PEF-PET mixtures) and 85% PEF up to slightly less than 100% PEF (e.g., 85–100 PEF in PET-PEF mixtures). The first derivative of heat flow as a function of temperature, depicted in Figure 4c can be also quite informative, as it shows dual *T*_g_s at nearly all compositions. However, the position of the low *T*_g_ shifts with composition especially for blends rich in PEF. This can be a signature of partial miscibility [41,42,43]. Based on those results we conclude that for 10% PET in the PET/PEF compositions, the blends appear to be miscible. Even more importantly, at the other end of the composition range, for PET/PEF compositions of 85/15 it appears that reasonable miscibility exists as well, while the 80/20 blend can be considered partially miscible.

Overall, from the above discussion it can be concluded that the window of miscibility can be observed for the PET- and PEF-rich polymer blends where the content of the rich phase is higher than 85%. Additionally, the 80–20 and 20–80 blends can be considered partially miscible.

### 3.2. Melting Behavior of the Blends

**Figure 5:**
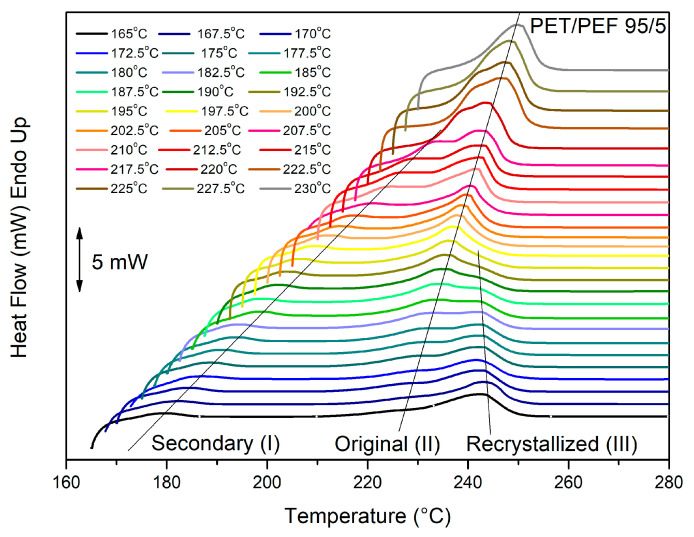
DSC heating scans at 20 °C/min for PET-PEF 95-5 blend samples after crystallization at the indicated temperature.

The melting behavior of the PET-PEF blends with high PET content was studied after isothermal crystallization from the melt at various temperatures. As it can be seen in Figure 5 for the example of the PET-PEF 95-5 blend, a multiple melting behavior characterized the materials. Similar behaviors have been observed in past for PET and related blends and in general for aromatic polyesters [44,45,46].

The multiplicity of the melting peaks is related with the isothermal crystallization temperatures. Heating following the crystallization at low temperatures (165 °C or lower for the PET-PEF 95-5) leads to dual melting peaks. For samples crystallized at temperatures above 170 °C but below 195 °C, triple melting was evidenced. For crystallization temperatures in the range between 195 and 205 °C, the ultimate temperature melting peak seemed to disappear, but a side peak was also present in place of the peak. Dual melting was observed after crystallization at *T_c_*s in the range 205 °C < *T_c_* < 227.5 °C. Finally, for *T_c_*s equal or above 230 °C the two peaks coincided, and a single melting peak was then observed. In all cases of thermograms of Figure 5 the low temperature peak (I) is the well-known annealing peak [47].

The middle temperature peak (II) can be attributed to the melting of the original crystals. However, this peak was not always well observed especially upon heating of samples crystallized at very low temperatures, because of the low degree of perfection and reduced thermal stability of the crystals. Finally, the ultimate temperature melting peak (III) was that corresponding to the melting of the reorganized/recrystallized material, that is for crystals stabilized via the crystal perfection processes upon heating.

### 3.3. Equilibrium Melting Point

The most popular method for the estimation of the equilibrium melting point of polymers is that of Hoffmann-Weeks (HF) [48]. According to this procedure, the measured melting temperatures (*T_m_*) of samples isothermally crystallized at various temperatures (*T_c_s*) are plotted against the crystallization temperatures. The linear extrapolation to the line *T_m_ = T_c_* gives an intercept equal to $T_{m}^{o}$. The associated equation is:

(1) $$T_{m}=T_{m}^{o}(1-\frac{1}{r})+\frac{T_{c}}{r}$$

where *T_m_* is the observed melting temperature of a crystal formed at a temperature *T_c_*, *r* is the thickening coefficient equal to *l_c_*/l_g_^*^ where *l_c_* is the thickness of the grown crystal and l_g_^*^ is the initial thickness of a chain-folded lamellar crystal [48]. The prerequisite for the application of this theory is the isothermal thickening process of lamellar crystals at a specific crystallization temperature and the dependence of the thickening coefficient on the crystallization temperature.

**Figure 6:**
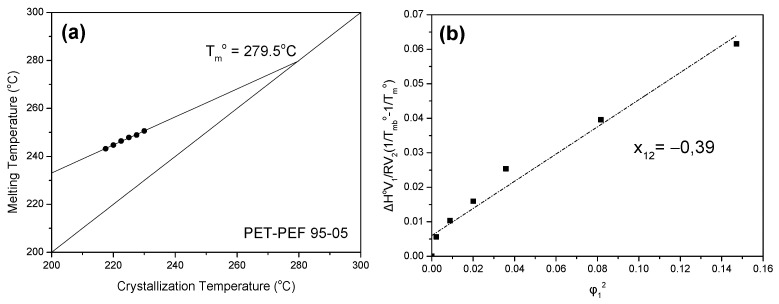
(**a**) Hoffman-Weeks plot for the determination of the equilibrium meting temperature of PET within the PET-PEF 95-05 blend, (**b**) Plots of $\frac{\Delta H^{0}V_{1}}{RV_{2}}\left(\frac{1}{T_{m(blend)}^{0}}-\frac{1}{T_{m(pure)}^{0}}\right)$. against $\phi_{1}^{2}$ using equilibrium melting temperatures.

The values for the temperature of the middle peak (II) were used to construct the Hoffman-Weeks plot for the PET-PEF blends with high PET content. The HW plot for PET-PEF 95-05 is shown in Figure 6a. Given the small variations in the melting temperatures of the blends we expect that the equilibrium melting temperature of the blends is in the vicinity of 280 °C.

The depression of the melting point of a crystalline polymer blended with an amorphous polymer provides information on the miscibility of the system [49]. In general, melting point depression is associated with morphological effects such as crystal thickness. Additionally, thermodynamic considerations predict that the chemical potential of a polymer will decrease with the addition of a (miscible) diluent. If the polymer is crystallizable, this decrease in chemical potential will result in a decreased equilibrium melting point. The equilibrium melting temperatures can be analyzed by the Flory-Huggins equation [50,51]:

(2) $$\frac{1}{T_{m(blend)}^{o}}-\frac{1}{T_{m(pure)}^{o}}=\frac{-R}{\Delta H^{0}}\frac{V_{2}}{V_{1}}\left[\frac{\ln\phi_{2}}{m_{2}}+\left(\frac{1}{m_{2}}-\frac{1}{m_{1}}\right)\phi_{1}+\chi_{12}\phi_{1}{}^{2}\right]$$

where the subscripts 1 and 2 refer to the amorphous and the crystalline polymer, respectively. $T_{m(pure)}^{o}$ and $T_{m(blend)}^{o}$ denote the equilibrium melting points of the pure crystallizable component and that of the blend, respectively. *V* is the molar volume of the repeating units of the polymers, *R* is the universal gas constant, Δ*H*^0^ is the heat of fusion of the perfectly crystallizable polymer, *m* is the degree of polymerization, ϕ is the volume fraction of the component in the blend, and *χ_12_* is the polymer-polymer interaction parameter. In fact, for the case of PET-PEF blends both polymers are semicrystalline. However, PEF has a lower melting temperature compared to PET (*T_m_*_(PEF)_ = 215 °C vs. *T_m_*_(PET)_ = 257.5 °C) as can be concluded from the DSC thermograms of Figure 2a. Thus, PEF remains in the liquid molten state at temperatures where PET can easily crystallize (215–230 °C). Based on this, PEF was assumed to be the amorphous component in its blends with PET. The molecular volume of PET is 143.82 cm^3^/mol and 127.27 cm^3^/mol for PEF, while the enthalpy of fusion is Δ*H_m_*^0^ = 26.9 kJ/mol for PEF [52].

For high molecular weight polymers, both *m*_1_ and *m*_2_ are large and the related terms can be neglected, thus Equation (2) takes the form:

(3) $$-\frac{\Delta H^{0}V_{1}}{RV_{2}}\left(\frac{1}{T_{m(blend)}^{0}}-\frac{1}{T_{m(pure)}^{0}}\right)=x_{12}\phi_{1}^{2}$$

If *χ*_12_ is assumed to be independent of composition, a plot of the left-hand side of Equation (3) versus $\phi_{1}^{2}$ should give a straight line passing through the origin. The interaction parameter is implicitly referred to a reference volume, *V_r_*, usually defined in terms of the molar volume of the amorphous component in the mixture. The interaction energy density is another form of expressing the Flory-Huggins interaction parameter, i.e., by eliminating the reference volume [53]:

(4) $$B=\frac{RTx_{12}}{V_{r}}$$

Substituting $\phi_{1}^{2}$ from this equation in the Flory-Huggins equation yields the Nishi- Wang equation [54]:

(5) $$T_{m}^{o}-T_{mb}^{o}=T_{m}^{o}\frac{BV_{2}}{\Delta H_{m}}\phi_{1}^{2}$$

In an attempt to calculate the polymer–polymer interaction parameter and to eliminate the morphological effect from the melting point depression, the equilibrium melting temperatures for pure PET and PET in the blends of given compositions were used (Figure 6b). The obtained values were *χ*_12_ = −0.39 and B= −3.37 cal/cm^3^. The negative *χ*_12_ and *B* values are consistent with a miscible system [55]. Additional insight on blend miscibility can be obtained by studying the crystallization kinetics by using both non-isothermal and purely isothermal conditions.

### 3.4. Crystallization of the Blends

#### 3.4.1. Isothermal Crystallization

**Figure 7:**
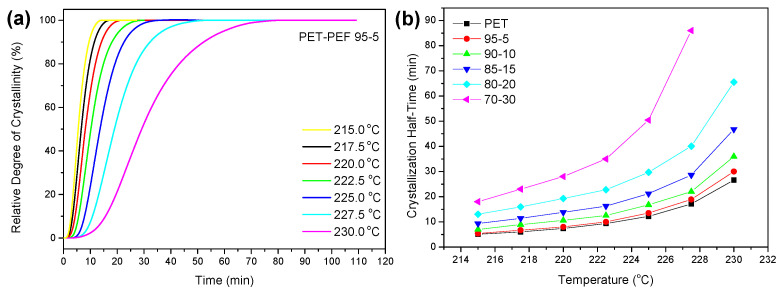
(**a**) Evolution of the relative degree of crystallinity with time for the PET-PEF 95-5 blend at various temperatures and (**b**) dependence of the isothermal crystallization half-time on the temperature for blends with high PET content.

The crystallization of polymer melts is most commonly accompanied by significant heat release. As a result, differential scanning calorimetry is the most accurate technique for the evaluation of the crystallization process. The isothermal crystallization of PET-PEF blends was studied at several crystallization temperatures and the exothermal curves were recorded as a function of time. The relative degree of crystallinity can be obtained if an assumption is made that the evolution of crystallinity is linearly proportional to the evolution of heat released during the crystallization:

(6) $$X(t)=\frac{\int_{0}^{t}(dH_{c}/dt)dt}{\int_{0}^{\infty }(dH_{c}/dt)dt}$$

where *dH_c_* denotes the measured enthalpy of crystallization during an infinitesimal time interval *dt*. The limits *t* and ∞ on the integrals are used to denote the elapsed time during the course of crystallization and at the end of the crystallization process, respectively. The evolution of the relative degree of crystallinity versus time for PET-PEF 95-5 blend can be observed in Figure 7a. The crystallization half-time can be used as a relative measure of the overall crystallization rate. Figure 7b shows the variation of the crystallization half-time (*t_1/2_*) values with crystallization temperature (*T_c_*). As it can be seen, the crystallization rates decrease with increasing crystallization temperature. In agreement with the results from the non-isothermal crystallization experiments there is a slow demixing process of PET/PEF blends in their melt state as a result of their partial miscibility. In order for PET (or PEF) to crystallize unlike segments will have to diffuse away to create sufficiently large domains for the nucleation and subsequent growth of PET crystals. 

#### 3.4.2. Application of Secondary Nucleation Theory

**Figure 8:**
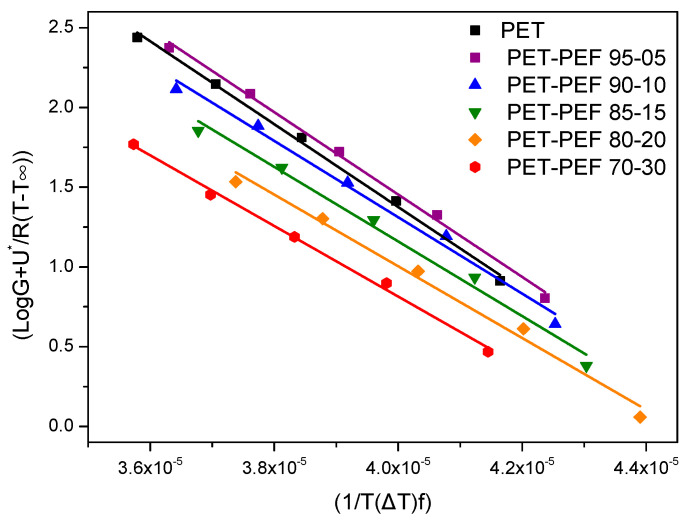
Lauritzen Hoffman-plots for PET and the blends.

The spherulite growth rate, *G*, as a function of temperature in isothermal crystallization can be described by the Lauritzen-Hoffman secondary nucleation theory [56,57,58] In this theory, there are two controlling temperatures; the equilibrium melting temperature and the liquid-to-glass temperature. Accordingly, *G* can be expressed as:

(7) $$G=G_{o}\exp\left[\frac{-U^{*}}{R(T_{c}-T_{\infty })}\right]\exp\left[\frac{-K_{g}}{T_{c}(\Delta T)f}\right]$$

where *G*_0_ is the pre-exponential factor, the first exponential term contains the contribution of diffusion process to the growth rate, while the second exponential term is the contribution of the nucleation process; U^*^ denotes the activation energy which characterizes molecular diffusion across the interfacial boundary between the melt and the crystal front, and *T*_∞_ is the “ideal” glass temperature located below the conventional *T*_g_. In a recent dielectric spectroscopy study the first term was extracted from the segmental dynamics of amorphous PEF with parameters, U* = 4.57 kcal/mol and *T*_∞_ = 289 K. *K_g_* is a nucleation constant and Δ*T* denotes the degree of undercooling (Δ*T* = *T_m_^0^* − *T*_c_); *f* is a correction factor which is close to unity at high temperatures and is given as *f = 2T_c_*/*(T_m_*^0^ + *T_c_*) [57]. Equation (7) can be also written in its logarithmic form and growth rate G, which should be obtained essentially by the spherulite growth, was calculated by the inverse of crystallization half time (1/*t*_1/2_). Actually, this substitution has been widely used in crystallization studies of polymers [57]. Then, plotting $\log G+\frac{U*}{2.30R(T_{c}-T_{\infty })}$ with respect to 1/(*T_c_*(Δ*T*)*f*) a straight line should appear, for each crystallization regime, having a slope equal to *K_g_*. Figure 8 gives the plot for the PET-PEF blends. From the slope and the intercept of the corresponding line, the nucleation parameter, *K_g_* and ln*G*_0_ can be obtained for each regime. The calculated values for *K_gII_* are summarized in Table 1.

For a secondary or heterogeneous nucleation, *K_g_* can be calculated from [57]:

(8) $$K_{g}=\frac{n\sigma \sigma_{e}b_{0}T_{m}^{0}}{\Delta h_{f}\rho_{c}k_{B}}$$

where, *n* is a constant equal to 4 for regime I and III and 2 for regime II, *σ*, and *σ_e_* are the side surface (lateral) and fold surface (end) free energies which measure the work required to create a new surface, *b*_0_ is the single layer thickness (0.595 nm for PET), Δ*h_f_ ρ_c_* = Δ*H_f_* is the enthalpy of melting per unit volume and was assumed to be Δ*H* = 2.04 × 10^8^ J/m^3^ for PET (given that the heat of fusion is 26.9 kJ/mol the density of the crystalline PET is 1.445 g/cm^3^) and *k_B_* is the Boltzmann constant (*k_B_* = 1.38 × 10^−23^ J/K). Below 236 and above 167 °C a crystallization regime II is assumed [59]. The resulting value for the product *σσ_e_* using *K_g_*_II_ = 2.6 × 10^5^ K^2^ was *σσ_e_* = 11.9 × 10^−4^ J^2^/m^4^. The results for the blends are summarized in Table 1. The lateral surface free energy, *σ* is commonly estimated as Equation (9):

(9) $$\sigma =\alpha \left(\Delta h_{f}\right){\left(a_{0}b_{0}\right)}^{0.5}$$

where, *α* was derived empirically to be 0.11 by analogy with the well-known behaviour of hydrocarbons. *a*_0_ and *b*_0_ factors are the monomolecular width and layer thickness, respectively. For PET *a*_0_ = 0.457 nm and *b*_0_ = 0.595 nm. Accordingly, *σ* = 1.09 × 10^−2^ J/m^2^. So, for PET *σ_e_* = 10.9 × 10^−2^ J/m^2^, a value that is close to previously reported ones [60,61].

Finally, the work of chain folding, *q*, which is most closely correlated with molecular structure, can be calculated from [62]:

(10) $$q=2a_{0}b_{0}\sigma_{e}$$

For PET it was found that *q* = 35.7 kJ/mol. The values for the blends were slightly lower indicating that the folding of macromolecular chains takes place easier in these samples, therefore facilitating the growth of crystals (Table 1).

#### 3.4.3. Non-Isothermal Crystallization

**Figure 9:**
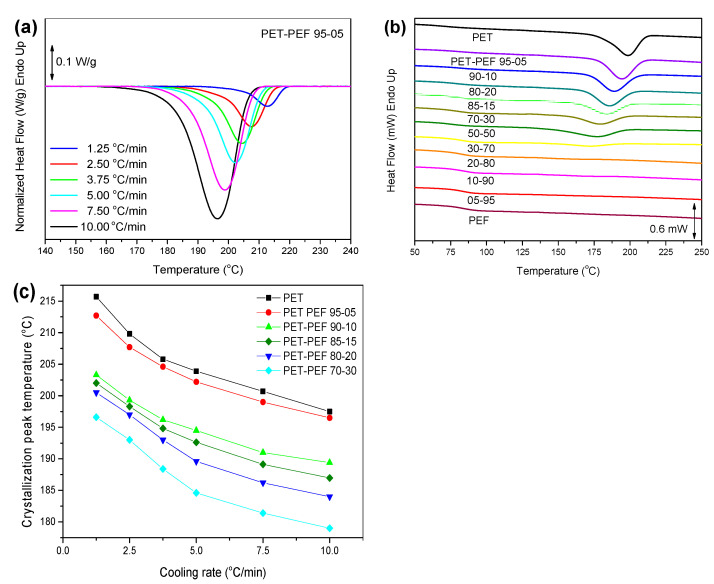
(**a**) DSC traces of non-isothermal crystallization of PET-PEF 95-05 at various cooling rates, (**b**) DSC traces of the non-isothermal crystallization of all blends under cooling at 10 °C/min, (**c**) peak crystallization temperatures versus cooling rate for the blends that were crystallized.

The non-isothermal crystallization of PET was investigated under several cooling rates, ranging from 1.25 to 10 °C/min (Figure 9a). The higher the cooling rate is, the broader the crystallization peaks. Furthermore, the increase of cooling rate leads to reduced peak temperatures, since the crystallization phenomenon starts and ends at higher times. From the data of the crystallization exotherms as a function of temperature *dH*_c_/*dT*, the relative crystallinity as a function of temperature can be calculated as follows [63,64,65]:

(11) $$X(T)=\frac{\int_{T_{0}}^{T_{c}}(dH_{c}/dT)dT}{\int_{T_{0}}^{T_{\infty }}(dH_{c}/dT)dT}$$

where *T*_0_ denotes the initial crystallization temperature and *T_c_* and *T*_∞_ the crystallization temperatures at time *t* and after the competition of the crystallization process, respectively. Additionally, once *X*(*T*) is obtained, the evolution of the relative degree of crystallinity can be plotted as a function of time, by using the time–temperature transformation: *t* = (*T*_0_ − *T*)/*a* [63,66]. The DSC curves from the non-isothermal crystallization at 10 °C/min for all blends are presented in Figure 9b. PET and the samples up to 30 wt.% PEF display the characteristic exothermic peaks, indicating the crystallization, while for PEF content higher than 30 wt.%, there is no clear crystallization. Finally, from the results of the peak temperature versus cooling rate for the crystallizable blends (Figure 9c), it can be seen that the characteristic peak crystallization temperatures decrease with increasing PEF content, independent of the cooling rate. This finding suggests that the demixing process of PET/PEF blends in their melt state is very slow and limited as a result of their partial miscibility.

### 3.5. Reactive Blending

**Figure 10:**
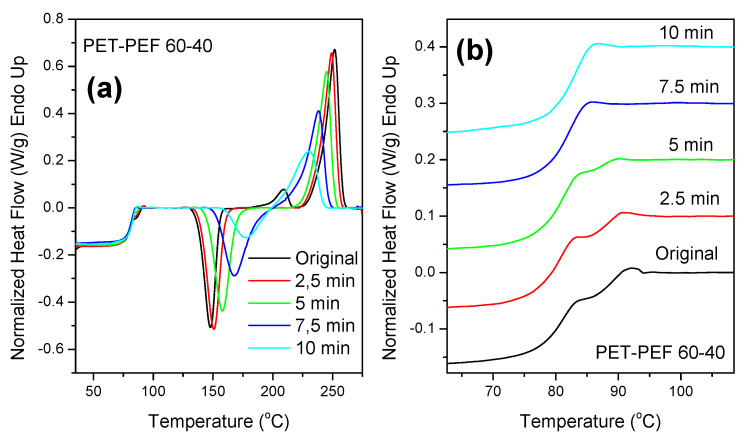
(**a**) Effect of blending time on the DSC curves of a PET-PEF 60-40 copolymer, (**b**) zoom-in of the glass transition temperature region of the blend at different blending times.

The use of random copolymers as compatibilizers has been established as an effective strategy to enhance miscibility in an otherwise immiscible (or partially miscible) blend. However, the formulation and preparation of a tailored random copolymer is a time-consuming process where precise steps need to be followed. For this reason, reactive blending is another process that can be followed for the preparation of miscible blends [67]. When two polyesters are mixed at temperatures above the melting point of at least one of them, transesterification reactions take place, which lead to the formation of block copolymers if the duration of mixing is long enough. In this work, we applied reactive blending for the PEF-PET 60-40 blends at 280 °C. The DSC curves of the blend at different reactive blending times can be seen in Figure 10a, while the glass transition region of interest can be seen in Figure 10b. Since a block copolymer is formed at increasing blending times, there is a suppression of cold-crystallization and a decrease of the melting temperatures. It is obvious that after 7.5 min of reactive blending the double glass transition of the blend is transformed to a single one, suggesting the formation of a block copolymer that is composed of dynamically homogeneous and thermodynamically miscible segments. 

### 3.6. Morphological Study of the Blends

**Figure 11:**
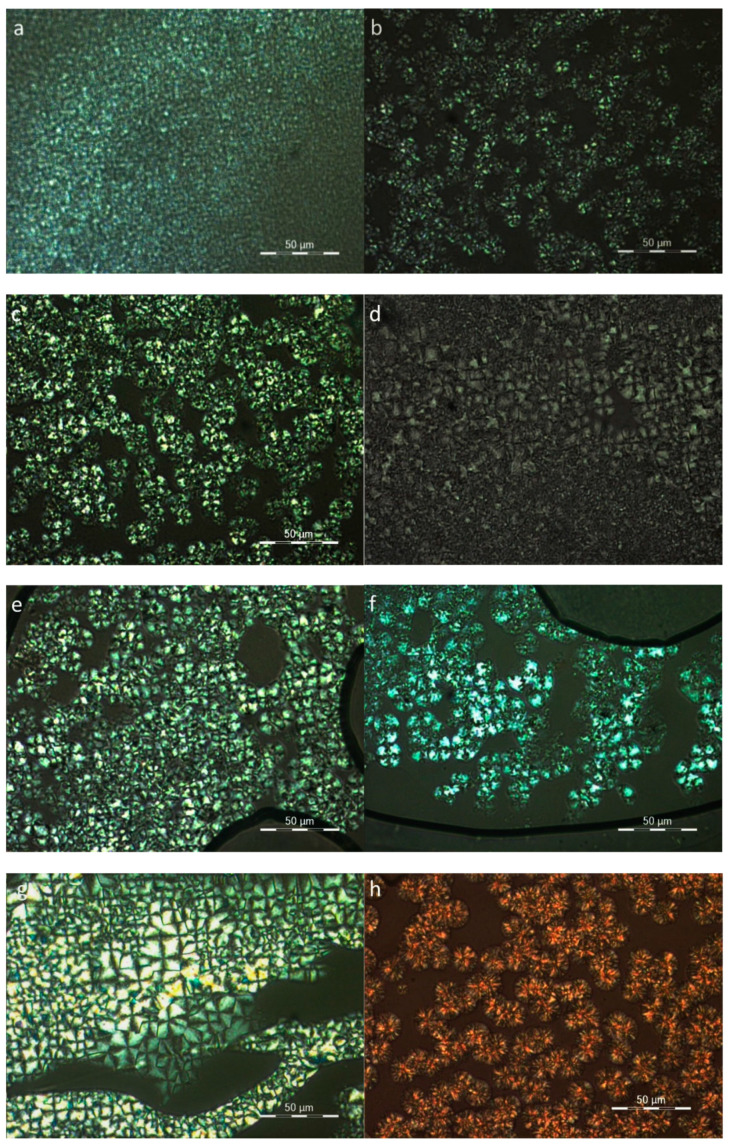
PLM photos recorded at 210 °C for the blends: (**a**) neat PET, (**b**) PET-PEF 90-10, (**c**) PET-PEF 80-20, (**d**) PET-PEF 60-40, (**e**) PET-PEF 50-50, (**f**) PET-PEF 40-60, (**g**) PET-PEF 30-70, (**h**) neat PEF.

The blend morphology was studied by means of polarized light microscopy under isothermal crystallization conditions at 210 °C. PET and PEF are two polyesters showing quite different morphologies upon isothermal crystallization. As can be seen in Figure 11a, PET crystallizes fast and, in any case, much faster than PEF. This might be the main limiting factor for miscibility in their blends. In the images presented in Figure 11 one can mainly see PET spherulites. This is because at 210 °C PEF crystallizes very slowly, so in the timescale of the experiments no PEF spherulites are expected to form. Moreover, in contrast to Figure 11a, the nucleation density of PET in the blends was reduced, so that well distinguishable spherulites formed, of significant diameter. Furthermore, the growth rates were reduced with increasing PEF content in the mixtures. 

## 4. Conclusions

Poly(ethylene terephthalate)/poly(ethylene 2,5-furandicarboxylate) blends have been successfully prepared by solution blending. The thermal transition temperatures and the thermodynamics of miscibility phenomena have been studied in detail by the use of advanced calorimetric techniques and measurement strategies. Miscibility was initially achieved at PET-rich (higher than 85%) and low-PET (lower than 15%) blends, while at intermediate compositions the blends were partially miscible. This conclusion has been exported from the study of both the glass transition temperatures of the blends and the Flory-Huggins interaction parameter. The equilibrium melting temperature of the PET within the PET-PEF 95-5 blend was obtained from the Hoffman-Weeks method and was found to be in the vicinity of 280 °C. Additionally, from the non-isothermal crystallization study, it was found that slow demixing process of PET/PEF blends takes place in their melt state as a result of their partial miscibility. Finally, reactive blending was also applied to enhance miscibility; after 7.5 min of mixing, the polymer blend was eventually transformed into a copolymer as concluded from the transformation of the dual glass transition to a single glass transition temperature.

The produced blends can be considered as excellent candidates to replace products and applications where PET is dominating the market against other biobased plastics. The presence of eco-friendly PEF, originating from renewable resources in the blends, enables a reduction of the environmental impact from the production of fully-PET based products while at the same time, the recyclability of the ultimate products remains high. Such blends are expected to play a major role in the near future for the increase in sustainability and the transition to green economies.

## Figures and Tables

**Table 1:** Results from the Lauritzen-Hoffman analysis on the crystallizable blends.

Blend	K_gII_ × 10^−5^ (K^2^)	*σσ_e_* × 10^4^ (J^2^/m^4^)	*σ* × 10^2^ (J/m^2^)	*σ_e_* × 10^2^ (J/m^2^)	q (kJ/mol)
PET	2.60	11.9	1.09	10.9	35.7
PET-PEF 95-05	2.58	11.8	1.09	10.8	35.4
PET-PEF 90-10	2.40	11.0	1.09	10.1	33.1
PET-PEF 85-15	2.34	10.7	1.09	9.85	32.3
PET-PEF 80-20	2.25	10.3	1.09	9.47	31.0
PET-PEF 70-30	2.21	10.2	1.09	9.37	30.7

## Data Availability

The data presented in this study are available on reasonable request from the corresponding author.

## References

[1] Sanders J.P.M., Clark J.H., Harmsen G.J., Heeres H.J., Heijnen J.J., Kersten S.R.A., Swaaij W.P.M., Moulijin J.A. (2012). Process intensification in the future production of base chemicals from biomass. Chem. Eng. Process..

[2] Zhang L., Jiang Y., Xiong Z., Liu X., Na H., Zhang R., Zhu J. (2013). Highly recoverable rosin-based shape memory polyurethanes. J. Mater. Chem. A.

[3] Storz H., Vorlop K.D. (2013). Bio-based plastics: Status, challenges and trends. Appl. Agric. For. Res..

[4] Mülhaupt R. (2012). Green Polymer Chemistry and Bio-based Plastics: Dreams and Reality. Macromol. Chem. Phys..

[5] Schneiderman D.K., Hillmyer M.A. (2017). 50th Anniversary Perspective: There Is a Great Future in Sustainable Polymers. Macromolecules.

[6] Gandini A., Lacerda T.M., Carvalho A.J.F., Trovatti E. (2016). Progress of Polymers from Renewable Resources: Furans, Vegetable Oils, and Polysaccharides. Chem. Rev..

[7] Lu J., Wu L., Li B.-G. (2017). High Molecular Weight Polyesters Derived from Biobased 1,5-Pentanediol and a Variety of Aliphatic Diacids: Synthesis, Characterization, and Thermo-Mechanical Properties. ACS Sustain. Chem. Eng..

[8] Sousa A.F., Vilela C., Fonseca A.C., Matos M., Freire C.S.R., Gruter G.-J.M., Coelho J.F.J., Silvestre A.J.D. (2015). Biobased polyesters and other polymers from 2,5-furandicarboxylic acid: A tribute to furan excellency. Polym. Chem..

[9] Morales-Huerta J.C., Ciulik C.B., De Ilarduya A.M., Muñoz-Guerra S. (2017). Fully bio-based aromatic–aliphatic copolyesters: Poly(butylene furandicarboxylate-co-succinate)s obtained by ring opening polymerization. Polym. Chem..

[10] Papageorgiou G.Z., Tsanaktsis V., Papageorgiou D.G., Exarhopoulos S., Papageorgiou M., Bikiaris D.N. (2014). Evaluation of polyesters from renewable resources as alternatives to the current fossil-based polymers. Phase transitions of poly(butylene 2,5-furan-dicarboxylate). Polymer.

[11] Papageorgiou G.Z., Tsanaktsis V., Papageorgiou D.G., Chrissafis K., Exarhopoulos S., Bikiaris D.N. (2015). Furan-based polyesters from renewable resources: Crystallization and thermal degradation behavior of poly(hexamethylene 2,5-furan-dicarboxylate). Eur. Polym. J..

[12] Zhang Z., Deng K. (2015). Recent Advances in the Catalytic Synthesis of 2,5-Furandicarboxylic Acid and Its Derivatives. ACS Catal..

[13] Rosenboom J.-G., Hohl D.K., Fleckenstein P., Storti G., Morbidelli M. (2018). Bottle-grade polyethylene furanoate from ring-opening polymerisation of cyclic oligomers. Nat. Commun..

[14] Burgess S.K., Wenz G.B., Kriegel R.M., Koros W.J. (2016). Penetrant transport in semicrystalline poly(ethylene furanoate). Polymer.

[15] Burgess S.K., Leisen J.E., Kraftschik B.E., Mubarak C.R., Kriegel R.M., Koros W.J. (2014). Chain Mobility, Thermal, and Mechanical Properties of Poly(ethylene furanoate) Compared to Poly(ethylene terephthalate). Macromolecules.

[16] Van Berkel J.G., Guigo N., Kolstad J.J., Sipos L., Wang B., Dam M.A., Sbirrazzuoli N. (2015). Isothermal Crystallization Kinetics of Poly (Ethylene 2,5-Furandicarboxylate). Macromol. Mater. Eng..

[17] Dimitriadis T., Bikiaris D.N., Papageorgiou G.Z., Floudas G. (2016). Molecular Dynamics of Poly(ethylene-2,5-furanoate) (PEF) as a Function of the Degree of Crystallinity by Dielectric Spectroscopy and Calorimetry. Macromol. Chem. Phys..

[18] Papageorgiou G.Z., Papageorgiou D.G., Terzopoulou Z., Bikiaris D.N. (2016). Production of bio-based 2,5-furan dicarboxylate polyesters: Recent progress and critical aspects in their synthesis and thermal properties. Eur. Polym. J..

[19] Tomisawa R., Ikaga T., Kim K., Ohkoshi Y., Okada K., Masunaga H., Kanaya T., Masuda M., Maeda Y. (2017). Effect of draw ratio on fiber structure development of polyethylene terephthalate. Polymer.

[20] Van Uytvanck P., Haire G., Marshall P., Dennis J. (2017). Impact on the Polyester Value Chain of Using p-Xylene Derived from Biomass. ACS Sustain. Chem. Eng..

[21] Galbis J.A., García-Martín M.D.G., De Paz M.V., Galbis E. (2016). Synthetic Polymers from Sugar-Based Monomers. Chem. Rev..

[22] Muñoz-Guerra S., Lavilla C., Japu C., De Ilarduya A.M. (2014). Renewable terephthalate polyesters from carbohydrate-based bicyclic monomers. Green Chem..

[23] Kim G.S., Son J.M., Lee J.K., Lee K.H. (2010). Morphology development and crystallization behavior of poly(ethylene terephthalate)/poly(trimethylene terephthalate) blends. Eur. Polym. J..

[24] Aoyama S., Park Y.T., Ougizawa T., Macosko C.W. (2014). Melt crystallization of poly(ethylene terephthalate): Comparing addition of graphene vs. carbon nanotubes. Polymer.

[25] Docampo P., Ball J.M., Darwich M., Eperon G.E., Snaith H.J. (2013). Efficient organometal trihalide perovskite planar-heterojunction solar cells on flexible polymer substrates. Nat. Commun..

[26] Kawai F., Kawabata T., Oda M. (2020). Current State and Perspectives Related to the Polyethylene Terephthalate Hydrolases Available for Biorecycling. ACS Sustain. Chem. Eng..

[27] Kim H.J., Peng X., Shin Y., Hillmyer M.A., Ellison C.J. (2021). Blend Miscibility of Poly(ethylene terephthalate) and Aromatic Polyesters from Salicylic Acid. J. Phys. Chem. B.

[28] Bai H., Xiu H., Gao J., Deng H., Zhang Q., Yang M., Fu Q. (2012). Tailoring impact toughness of poly (L-lactide)/poly (ε-caprolactone)(PLLA/PCL) blends by controlling crystallization of PLLA matrix. ACS Appl. Mater. Interfaces.

[29] Doroshenko M., Gonzales M., Best A., Butt H.-J., Koynov K., Floudas G. (2012). Monitoring the Dynamics of Phase Separation in a Polymer Blend by Confocal Imaging and Fluorescence Correlation Spectroscopy. Macromol. Rapid Commun..

[30] Blochowiak M., Pakula T., Butt H.-J., Floudas G. (2007). Miscibility of binary blends of ethylene/norbornene copolymers: Comparison to a lattice cluster theory. Polymer.

[31] Suzuki T., Tanaka H., Nishi T. (1989). Miscibility and transesterification in bisphenol A polycarbonate/poly(ethylene terephthalate) blends. Polymer.

[32] Daubeny R.D.P., Bunn C. (1954). The crystal structure of polyethylene terephthalate. Proc. Royal.

[33] Mao Y., Kriegel R.M., Bucknall D.G. (2016). The crystal structure of poly(ethylene furanoate). Polymer.

[34] Tsanaktsis V., Papageorgiou D.G., Exarhopoulos S., Bikiaris D.N., Papageorgiou G.Z. (2015). Crystallization and Polymorphism of Poly(ethylene furanoate). Cryst. Growth Des..

[35] Lotti N., Munari A., Gigli M., Gazzano M., Tsanaktsis V., Bikiaris D.N., Papageorgiou G.Z. (2016). Thermal and structural response of in situ prepared biobased poly(ethylene 2,5-furan dicarboxylate) nanocomposites. Polymer.

[36] Papageorgiou G.Z., Papageorgiou D.G., Chrissafis K., Bikiaris D., Will J., Hoppe A., Roether J.A., Boccaccini A.R. (2013). Crystallization and Melting Behavior of Poly(Butylene Succinate) Nanocomposites Containing Silica-Nanotubes and Strontium Hydroxyapatite Nanorods. Ind. Eng. Chem. Res..

[37] Stoclet G., du Sart G.G., Yeniad B., de Vos S., Lefebvre J. (2015). Isothermal crystallization and structural characterization of poly(ethylene-2,5-furanoate). Polymer.

[38] Martino L., Guigo N., Van Berkel J.G., Sbirrazzuoli N. (2017). Influence of organically modified montmorillonite and sepiolite clays on the physical properties of bio-based poly(ethylene 2,5-furandicarboxylate). Compos. Part B Eng..

[39] Xie H., Meng H., Wu L., Li B.G., Dubois P. (2019). In-situ synthesis, thermal and mechanical properties of biobased poly (ethylene 2, 5-furandicarboxylate)/montmorillonite (PEF/MMT) nanocomposites. Eur. Polym. J..

[40] Woo E.M., Kuo Y.-H. (2003). Complete miscibility of ternary aryl polyesters demonstrating a new criterion and horizon for miscibility characterization. J. Polym. Sci. Part B Polym. Phys..

[41] Lodge T.P., McLeish T.C.B. (2000). Self-Concentrations and Effective Glass Transition Temperatures in Polymer Blends. Macromolecules.

[42] Lodge T.P., Wood E.R., Haley J.C. (2006). Two calorimetric glass transitions do not necessarily indicate immiscibility: The case of PEO/PMMA. J. Polym. Sci. Part B Polym. Phys..

[43] Kordjazi Z., Ajji A. (2020). Partially miscible polymer blends of ethyl cellulose and hydroxyl terminated polybutadiene. Polymer.

[44] Papageorgiou G.Z., Tsanaktsis V., Bikiaris D.N. (2014). Synthesis of poly(ethylene furandicarboxylate) polyester using monomers derived from renewable resources: Thermal behavior comparison with PET and PEN. Phys. Chem. Chem. Phys..

[45] Chen M.-J., Shi Q.-S. (2015). Transforming Sugarcane Bagasse into Bioplastics via Homogeneous Modification with Phthalic Anhydride in Ionic Liquid. ACS Sustain. Chem. Eng..

[46] Minakov A.A., Mordvintsev D.A., Schick C. (2004). Melting and reorganization of poly(ethylene terephthalate) on fast heating (1000 K/s). Polymer.

[47] Zhou C., Clough S.B. (1988). Multiple melting endotherms of poly(ethylene terephthalate). Polym. Eng. Sci..

[48] Hoffman J.D., Weeks J.J. (1962). Melting process and the equilibrium melting temperature of polychlorotrifluoroethylene. J. Res. Natl. Bur. Stand. Sect. A Phys. Chem..

[49] Runt J., Miley D.M., Zhang X., Gallagher K.P., McFeaters K., Fishburn J. (1992). Crystallization of poly(butylene terephthalate) and its blends with polyarylate. Macromolecules.

[50] Flory P.J. (1949). Thermodynamics of Crystallization in High Polymers. IV. A Theory of Crystalline States and Fusion in Polymers, Copolymers, and Their Mixtures with Diluents. J. Chem. Phys..

[51] Flory P.J. (1955). Theory of crystallization in copolymers. Trans. Faraday Soc..

[52] Konstantopoulou M., Terzopoulou Z., Nerantzaki M., Tsagkalias J., Achilias D.S., Bikiaris D.N., Exarhopoulos S., Papageorgiou D.G., Papageorgiou G.Z. (2017). Poly(ethylene furanoate-co-ethylene terephthalate) biobased copolymers: Synthesis, thermal properties and cocrystallization behavior. Eur. Polym. J..

[53] Meaurio E., Sanchez-Rexach E., Hernandez E.Z., Lejardi A., Sanchez-Camargo A.D.P., Sarasua J.-R. (2017). Predicting miscibility in polymer blends using the Bagley plot: Blends with poly(ethylene oxide). Polymer.

[54] Qiu Z., Komura M., Ikehara T., Nishi T. (2003). Poly (butylene succinate)/poly (vinyl phenol) blends. Part 1. Miscibility and crystallization. Polymer.

[55] Penning J.P., Manley R.S.J. (1996). Miscible Blends of Two Crystalline Polymers. 1. Phase Behavior and Miscibility in Blends of Poly(vinylidene fluoride) and Poly(1,4-butylene adipate). Macromolecules.

[56] Lauritzen J.I., Hoffman J.D. (1959). Formation of polymer crystals with folded chains from dilute solution. J. Chem. Phys..

[57] Hoffman J.D., Miller R.L. (1997). Kinetic of crystallization from the melt and chain folding in polyethylene fractions revisited: Theory and experiment. Polymer.

[58] Medellín-Rodríguez F.J., Phillips P.J., Lin J.S. (1995). Application of Secondary Nucleation Theory to Semirigid Macromolecules: PEEK, PET, and PEN. Macromolecules.

[59] Rahman M., Nandi A.K. (2002). On the crystallization mechanism of poly(ethylene terepthalate) in its blends with poly(vinylidene fluoride). Polymer.

[60] Lu X., Hay J. (2001). Isothermal crystallization kinetics and melting behaviour of poly(ethylene terephthalate). Polymer.

[61] Papageorgiou G.Z., Karandrea E., Giliopoulos D.J., Papageorgiou D.G., Ladavos A., Katerinopoulou A., Achilias D.S., Triantafyllidis K.S., Bikiaris D.N. (2014). Effect of clay structure and type of organomodifier on the thermal properties of poly(ethylene terephthalate) based nanocomposites. Thermochim. Acta.

[62] Thomas D.G., Staveley L.A.K. (1952). 889. A study of the supercooling of drops of some molecular liquids. J. Chem. Soc..

[63] Jeziorny A. (1978). Parameters characterizing the kinetics of the non-isothermal crystallization of poly(ethylene terephthalate) determined by d.s.c. Polymer.

[64] Di Lorenzo M., Silvestre C. (1999). Non-isothermal crystallization of polymers. Prog. Polym. Sci..

[65] Guigo N., van Berkel J., de Jong E., Sbirrazzuoli N. (2017). Modelling the non-isothermal crystallization of polymers: Application to poly (ethylene 2, 5-furandicarboxylate). Thermochim. Acta.

[66] Avrami M. (1939). Kinetics of phase change. I General theory. J. Chem. Phys..

[67] Formela K., Zedler Ł., Hejna A., Tercjak A. (2018). Reactive extrusion of bio-based polymer blends and composites—Current trends and future developments. Express Polym. Lett..

